# Clipless management of the renal vein during hand-assist laparoscopic donor nephrectomy

**DOI:** 10.1186/1471-2490-6-23

**Published:** 2006-09-15

**Authors:** Gregory S Rosenblatt, Michael J Conlin

**Affiliations:** 1 Department of Surgery, Division of Urology and Renal Transplantation, Oregon Health & Science University, 3181 Sam Jackson Park Road, Mail code L588, Portland, Oregon, 97201, USA

## Abstract

**Background:**

Laparoscopic live donor nephrectomy has become the preferred method of donor nephrectomy at many transplant centers. The laparoscopic stapling device is commonly used for division of the renal vessels. Malfunction of the stapling device can occur, and is often due to interference from previously placed clips. We report our experience with a clipless technique in which no vascular clips are placed on tributaries of the renal vein at or near the renal hilum in order to avoid laparoscopic stapling device misfires.

**Methods:**

From December 20, 2002 to April 12, 2005, 50 patients underwent hand-assisted laparoscopic left donor nephrectomy (LDN) at our institution. Clipless management of the renal vein tributaries was used in all patients, and these vessels were divided using either a laparoscopic stapling device or the LigaSureTM device (Valleylab, Boulder, CO). The medical and operative records of the donors and recipients were reviewed to evaluate patient outcomes.

**Results:**

The mean follow-up time was 14 months. Of the 50 LDN procedures, there were no laparoscopic stapling device malfunctions and no vascular complications. All renal allografts were functioning at the time of follow-up.

**Conclusion:**

Laparoscopic stapling device failure due to deployment across previously placed surgical clips during laparoscopic live donor nephrectomy can be prevented by not placing clips on the tributaries of the renal vein. In our series, there were no vascular complications and no device misfires. We believe this clipless technique improves the safety of laparoscopic donor nephrectomy.

## Background

Since the first reported laparoscopic nephrectomy in 1991, laparoscopic technique for kidney surgery has rapidly gained acceptance. Laparoscopic live donor nephrectomy has become the preferred method of donor nephrectomy at many transplant centers [1]. Compared to open donor nephrectomy, laparoscopic surgery offers the advantage of quicker recovery and a smaller incision. The laparoscopic stapling device is commonly used for division of the renal vessels. Malfunction of the laparoscopic stapling device can occur, and is often due to interference from previously placed clips. In one published study, 50% of misfires were associated with deployment of the device over previous surgical clips [2]. We report our experience with a clipless technique in which no vascular clips are placed on tributaries of the renal vein at or near the renal hilum in order to avoid laparoscopic stapling device misfires. We consider this technique to be clipless even when a stapling device is deployed across a previously placed line of staples. Contrary to the misfires that occur when a staple line is deployed over vascular clips, deployment of the stapling device over a previous staple line is acceptable and should not result in a misfire or malfunction.

## Methods

**Figure 1:**
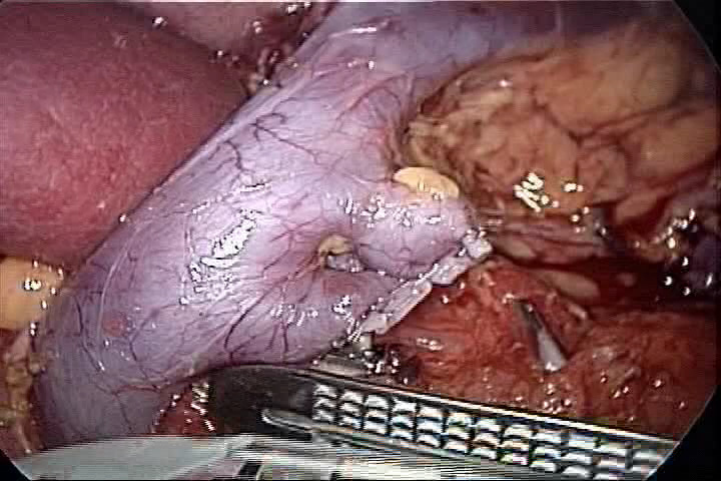
**Division of the renal vein**. Clipless technique in which no vascular clips are placed on tributaries of the renal vein at or near the renal hilum. Deployment of the stapling device over a previous staple line is acceptable and should not result in a misfire or malfunction.

We obtained protocol approval from the Research Development & Administration Institutional Review Board (IRB) at Oregon Health & Science University before retrospectively reviewing the records of 50 consecutive patients who underwent hand-assisted left laparoscopic live donor nephrectomy and their corresponding kidney transplant recipients. The surgeries took place at our institution between December 20, 2002 and April 12, 2005. Clipless management of the renal vein tributaries was used in all patients, and this involved division of the tributaries near the renal vein using the LigaSureTM device for vessels 7 mm in diameter or less, or using the laparoscopic stapling device for larger vessels. We performed bench ligation of stumps controlled with LigaSure. A single surgeon (MC) was the primary surgeon for all cases. The Endo-GIA (US Surgical, Norwalk, CT) with a 2.0 mm articulating vascular staple load was used to divide the renal vein in 46 patients (Figure 1). The Endo-TA stapler (US Surgical, Norwalk, CT) was used in 4 patients. The medical and operative records of the donors and recipients were reviewed to evaluate patient outcome.

## Results

The mean patient age was 42 years (range 21.5 to 64.7) and consisted of 19 men and 31 women. The mean preoperative donor serum creatinine value was 0.82 mg/dL (range 0.6 to 1.3). The mean warm ischemia time was 123 seconds (range 50 to 240), and the mean cold ischemia time was 137 minutes (range 11 to 314). The mean number of tributaries draining into the renal vein was 3 (range 1 to 6). These included lumbar, gonadal, and adrenal veins. The mean operative time (incision until out of operating room) was 3.6 hours (range 2.6 to 5.2). Of the 50 LDN, there were no laparoscopic stapling device malfunctions and no vascular complications. Minor postoperative complications occurred in two patients, both of whom developed an ipsilateral grade 3 varicocele. These both resolved without additional treatment. At one-month post-op, the mean donor creatinine values was 1.24 mg/dL (range 0.9 to 1.8). At a mean follow-up time of 14.2 months (range 1–29) all renal allografts were functioning normally.

## Discussion

Laparoscopic donor nephrectomy has been shown to be a safe alternative to the open surgical approach [1,3-7]. Techniques to manage the renal vein without clips have been reported, and include bipolar electrocoagulation [8.9]. We commonly use the LigaSureTM device, which works via a feedback-controlled response system that automatically discontinues energy delivery at the completion of vessel fusion [10]. This minimizes thermal spread and helps to avoid charring, which can cause the instrument to stick to the cauterized vessel. The manufacturers recommend 7 mm as the upper limit of vessel diameter that should be controlled with their device.

The use of an endoscopic linear stapling device at the renal hilum has become standard. Risks associated with misfire of the laparoscopic stapling device include significant vascular injury and intraoperative bleeding. This may require conversion to open nephrectomy in order to control bleeding, resulting in increased warm ischemia time and greater patient morbidity. Using bipolar electrocautery alone to cauterize renal vein branches, Schuster and Wolf reported easier stapler application and a decrease in average warm ischemia time, which was not statistically significant [9].

A 1.7% rate of stapling device malfunction has been reported during laparoscopic nephrectomy, and 50% of the stapling device misfires were due to deployment over a previously placed surgical clip near the renal hilum [2]. Use of this clipless technique should decrease the laparoscopic stapling device misfire rate by one-half.

An additional benefit to the clipless technique is that in many cases the laparoscopic stapling device can be fired medial to the left adrenal vein, allowing for procurement of a longer left renal vein.

## Conclusion

Laparoscopic stapling device failure due to deployment across previously placed surgical clips during laparoscopic live donor nephrectomy can be prevented by not placing clips on the tributaries of the renal vein. In our series of 50 consecutive patients, the left kidney was procured by hand-assist laparoscopic technique, and there were no vascular complications and no device misfires. We have since utilized this technique safely during both right and left laparoscopic donor nephrectomy, and we believe this improves the safety of laparoscopic donor nephrectomy.

## Abreviations

LDN = Laparoscopic Donor Nephrectomy

MC = Michael Conlin

## Competing interests

The author(s) declare that they have no competing interests.

## Authors' contributions

Both authors provided significant contribution to the design, research, and preparation of this manuscript.

## Pre-publication history

The pre-publication history for this paper can be accessed here:
